# Prevalence of anogenital HPV infection, related disease and risk factors among HIV-infected men in inner-city Johannesburg, South Africa: baseline findings from a cohort study

**DOI:** 10.1186/s12889-017-4354-0

**Published:** 2017-07-04

**Authors:** Admire Chikandiwa, Lucy Chimoyi, Pedro T Pisa, Matthew F Chersich, Etienne E Muller, Pamela Michelow, Philippe Mayaud, Sinead Delany-Moretlwe

**Affiliations:** 1 Wits Reproductive Health and HIV Institute (WRHI), Faculty of Health Sciences, University of the Witwatersrand, Johannesburg, South Africa; 2 National Institute for Communicable Diseases, National Health Laboratory Service, Johannesburg, South Africa; 3 Department of Anatomical Pathology, Cytology Unit, University of the Witwatersrand, Johannesburg, South Africa; 4 London School of Hygiene and Tropical Medicine, London, UK

**Keywords:** Anogenital infection, Human papillomavirus, Anogenital warts, Anal cytology, HIV, Men, South Africa

## Abstract

**Background:**

Persistent high-risk human papillomavirus (HR-HPV) infection is associated with the development of anogenital cancers, particularly in men living with HIV (MLWH). We describe the prevalence of anogenital HPV infection, abnormal anal cytology and anogenital warts (AGWs) in MLWH in Johannesburg, and explore whether HPV infection and receipt of antiretroviral treatment is associated with detection of abnormal anal cytology and AGWs.

**Methods:**

We enrolled a cohort of 304 sexually-active MLWH ≥18 years, who completed a questionnaire and physical examination. Genital swabs were collected from all men and intra-anal swabs from 250 (82%). Swabs were tested for HPV DNA and genotypes, and anal smears graded using the Bethesda classification. Factors associated with anogenital disease were assessed by logistic regression models.

**Results:**

Two thirds were receiving antiretroviral treatment, for a median 33 months (IQR = 15–58) and 54% were HIV-virologically suppressed. Only 5% reported ever having sex with men. Among 283 genital swabs with valid results, 79% had any HPV, 52% had HR-HPV and 27% had >1 HR-HPV infection. By comparison, 39% of the 227 valid intra-anal swabs had detectable HPV, 25% had any HR-HPV and 7% >1 HR infection. While most anal smears were normal (51%), 20% had ASCUS and 29% were LSIL. No cases had HSIL or cancer. Infection with >1 HR type (adjusted OR [aOR] = 2.39; 95%CI = 1.02–5.58) and alpha-9 types (aOR = 3.98; 95%CI = 1.42–11.16) were associated with having abnormal cytology. Prevalence of AGWs was 12%. Infection with any LR type (aOR = 41.28; 95%CI = 13.57–125.62), >1 LR type (aOR = 4.14; 95%CI = 1.60–10.69), being <6 months on antiretroviral treatment (aOR = 6.90; 95%CI = 1.63–29.20) and having a CD4+ count <200 cells/μL (aOR = 5.48; 95%CI: 1.60–18.78) were associated with having AGWs.

**Conclusions:**

In this population, anogenital HR-HPV infection and associated low-grade disease is common, but severe anal dysplasia was not detected. Findings reinforce the need for HPV vaccination in men for preventing both AGWs and HR-HPV infection. Given the absence of anal HSILs, however, the findings do not support the use of anal screening programmes in this population.

**Electronic supplementary material:**

The online version of this article (doi:10.1186/s12889-017-4354-0) contains supplementary material, which is available to authorized users.

## Background

Anogenital human papillomavirus (HPV) is the most frequent sexually transmitted infection (STI) in the world, in both men and women [1, 2]. The burden of anogenital HPV infections in men, as measured by HPV DNA prevalence, ranges widely, from estimates of 2 to 84% [3]. Anogenital HPV is classified into high-risk (HR) types, that are implicated in men in the evolution of anal and penile cancers, and low-risk (LR) types which are largely associated with the development of anogenital warts (AGWs) [4]. An estimated 20–30% of men with anogenital HPV infection are reported to develop persistent HPV infection that can progress to anogenital cancers and/or warts [5, 6]. Persistent infection with the HR types 16 and 18 is believed to be responsible for at least 80% of anal cancer cases and its precursor, anal intraepithelial neoplasia (AIN) [7, 8]. The LR-HPV types 6 and 11 are the predominant cause of AGWs [9, 10]. While AGWs are benign, they are a source of psychosocial distress [11] and can cause physical discomfort, including bleeding and itching [12]. Approximately 25% of AGWs will spontaneously regress [12], however, recurrence is common, resulting in high medical costs from repeated treatment [13].

Although data for men in sub-Saharan Africa (SSA) are limited, overall reported anogenital HPV DNA prevalence is high, ranging from 19 to 78% [14–19]. The incidence of anogenital HR-HPV infection in men in South Africa is also concerning, averaging 40 per 100 person-years [20–22]. HIV may alter the susceptibility to, and natural history of, HPV-associated diseases [23, 24]. Men living with HIV (MLWH) are at increased risk for prevalent anogenital HPV infection and anogenital cancers, regardless of sexual behaviour [24, 25]. There is evidence that AGWs in immunocompromised MLWH have a prolonged clinical course [26, 27]. This is in part attributed to immune dysfunction which impairs immuno-surveillance and clearance of viral infections such as HPV [24, 28]. While data suggest that the progression of AIN disease is less predictable than cervical cancer with some AIN lesions spontaneously regressing, [6] ART does not appear to reverse the risk for anal cancer once AIN is identified [29].

A number of strategies have been proposed to reduce anogenital HPV infection and its associated burden of disease in men. Screening for AIN using anal cytology has been recommended in some high-income countries [30]. However, the benefits for screening for AIN in low- to middle-income countries (LMICs), such as South Africa, are uncertain. HPV vaccines, used as primary prevention, have been shown to be efficacious in women, and vaccine studies have now demonstrated evidence of benefit in men [31].

While HPV vaccination programmes for girls residing in LMICs have scaled up [32], questions remain about whether to include boys or to target vaccination to high-risk groups of women and men, such as MLWH. Decisions about vaccination in MLWH, who constitute a large population in many LMICs, are contingent on the epidemiology of HPV infection and related disease in men [33]. Using data collected from MLWH who were recruited into a cohort in inner-city Johannesburg, we estimated the prevalence of anogenital HPV infection and associated anogenital disease, as well as the uptake of anal canal swabbing. We further examined the relationships between HPV infections, antiretroviral treatment, CD4 count and HIV viral load, and the presence of either abnormal anal cytology or AGWs. Finally, we explored the implications of the study findings for HPV prevention.

## Methods

### Study design, setting and population

We analysed baseline data from MLWH enrolled in a prospective cohort study. The primary aim of the cohort study was to evaluate the natural history of HPV infection and disease in HIV-infected men in South Africa to help inform the selection of HPV prevention interventions in this population, such as primary or secondary HPV vaccination, and anal screening. In inner-city Johannesburg, between October 2012 and August 2013, men in the community or attending HIV clinics were approached and asked to volunteer for the study. Potential participants were screened for eligibility and enrolled if they were willing to provide informed consent, 18 years or older, reported at least one episode of sexual intercourse in the past 3 months and were HIV positive on rapid testing. All procedures were conducted at a research clinic in downtown Johannesburg.

### Questionnaire administration and clinical procedures

Participants were interviewed by male nurses using a structured questionnaire that covered socio-demographic, medical and behavioural characteristics. For questions on sensitive topics, participants were given a tablet computer to self-complete data collection. The participants, all of whom had completed at least a primary education, were given a demonstration from the research nurse on how to use the tablet. A nurse or doctor conducted a physical examination to determine the presence of AGWs and circumcision status. The anatomical location of warts was marked on a diagram of the genitalia. Digital rectal examination was performed on all participants to exclude gross abnormalities. One participant with a palpable abnormality was referred to a colorectal surgeon for further assessment and management. The final diagnosis was anal warts. Blood samples were then collected and stored at 8 °C before testing for CD4+ count and HIV plasma viral load (PVL). Participants who had a CD4+ count <350 cells/μL were referred to an HIV clinic for ART initiation, in accordance with the national guidelines at the time [34].

Clinicians were trained in intra-anal swab collection by a colorectal surgeon; competency was established in practical sessions that involved ten men. Smears collected during the training sessions were subsequently assessed by a cytopathologist at the National Health Laboratory Service (NHLS) as having adequate cellular material for analysis.

Genital HPV DNA was assessed on a cotton swab that was rubbed gently, but firmly, around the glans penis, coronal sulcus, and then down the ventral surface of the penis and the scrotum [35]. Two intra-anal swabs were collected by blindly inserting a saline-moistened dacron swab 3 cm into the anal canal, and then removing it whilst rotating the swab and applying gentle pressure on the walls of the canal [4]. One intra-anal swab was used for HPV DNA testing, while the other was used to prepare a conventional anal smear for cytological analysis, by rolling the swab on a glass side and then fixing it with cytological fixative spray. Participants were informed that they could opt out of any procedures they felt uncomfortable with. Swabs were stored at -70 °C before testing.

### Laboratory testing

HIV status was assessed by two rapid tests (Alere Determine™ HIV-1/2 [Alere International Limited, Galway, Ireland] and Uni-Gold™ Recombigen® HIV-1/2 [Trinity Biotech PLC, Co. Wicklow, Ireland]), in accordance with national guidelines [36]. Among those that tested positive, CD4+ count was measured using FACScount, BD™ (BD Biosciences, United States). HIV RNA was assessed by Roche Taqman® (Roche Diagnostics, Mannheim, Germany).

Identical methods of HPV testing were used for genital and intra-anal swabs*.* DNA was extracted from swabs using the MagNA Pure LC DNA Isolation Kit I in combination with the MagNA Pure LC 2.0 automated nucleic acid extractor (Roche Diagnostics, Mannheim, Germany). Thereafter, HPV genotype distributions were assessed using the Roche Linear Array (Roche Diagnostics, Mannheim, Germany). The GeneAmp 9700 PCR System (Roche Diagnostics, Mannheim, Germany) was used to amplify the target HPV DNA for 37 anogenital HPV genotypes, including both LR HPV types (6, 11, 26, 40, 42, 53, 54, 55, 61, 62, 64, 66, 67, 69, 70, 71, 72, 73, 81, 82, 83, 84, IS39 and CP6108) and HR HPV types (16, 18, 31, 33, 35, 39, 45, 51, 52, 56, 58, 59 and 68). HR HPV types were defined using the current International Agency for Research on Cancer (IARC) classification [37]. The assay included a β-globin human gene target to control for cell adequacy, extraction and amplification. All procedures were performed in accordance with manufacturer’s instructions.

Conventional anal smears were examined as a single batch at the end of the study. Anal smears were stained by the Papanicolaou stain before being analysed and classified according to the Bethesda System (liquid-based cytology is not available within the South African public health care system and thus was not used) [38]. Smears were graded as either unsatisfactory for analysis, negative for intraepithelial malignancy (NILM), atypical squamous cells of undetermined significance (ASCUS), atypical squamous cells-high grade lesions cannot be ruled out (ASC-H), and low- (LSIL) or high-grade squamous intraepithelial lesions (HSIL). Smears were read independently by a cytotechnologist and one cytopathologist at the NHLS in Johannesburg. If there was a discrepancy between these two readings, the smear was given to a second cytopathologist. Grade ASCUS or higher was considered abnormal.

### Study variables and analysis

Participants reporting always using a condom with their most recent partner were classified as consistent condom users. MLWH were defined as having controlled HIV disease if they had been receiving ART for at least 6 months, an undetectable PVL (plasma HIV RNA <40 copies/ml) and a CD4+ count greater than 350 cells/μL. Two additional variables for HPV infection were created, namely: any alpha-7 category (HPV types 18, 39, 45 and 59); any alpha-9 category (HPV types 16, 31, 33, 35, 52 and 58). To assess uptake of anal swabbing, participants were specifically asked if they were willing to have anal swabbing before the intra-anal swabs were taken and the response was noted on the study case report form.

Data were summarized using frequencies, means (standard deviation [SD]), or medians (interquartile range [IQR]). Student *t* and Mann-Whitney tests for continuous variables and chi-squared tests for categorical variables were used to detect differences in characteristics between those with and without HPV DNA. Logistic regression models were used to explore whether HPV infection status and antiretroviral treatment status, CD4 count and HIV viral load were associated with having the two HPV disease outcomes (any anal cytological abnormality and AGWs). The following three models were computed, M1: bivariate (crude); M2: adjusted for age; and M3 adjusted for age, CD4+ count, duration on ART, circumcision status (for AGWs only) and marital status (for abnormal cytology only) [39, 40]. All analyses were performed in Stata™ Version 12 [41].

## Results

### Participant characteristics

A total of 304 men with a mean age of 38 years (SD, 8 years) were enrolled between March 2011 and October 2012 (Table 1). The majority of participants were born in South Africa (68%) and unmarried (59%), with 25% reporting more than one sexual partner in the previous 3 months. Only 5% (*n* = 15) reported ever having sex with other men. Of these, 10 reported that they were MSM and had had receptive anal sex, while the other 5 had never had receptive anal sex. A third (31%) were cigarette smokers and 20% circumcised. Most participants (65%) were already taking ART (*n* = 197), for a median duration of 33 months (IQR, 15–58). Among those on ART, 54% were virologically suppressed, with a median CD4+ count of 445 cells/μL (IQR, 328–567) and only 9% had a CD4+ count <200 cells/μL.

**Table 1:** Characteristics of participants, by genital HPV DNA status

Characteristic	HPV DNA detected *N* = 224 n (%) or Mean (SD) or Median (IQR)	Total *N* = 304 n (%) or Mean (SD) or Median (IQR)
Age, mean years	38 (8)	38 (8)
Born in SA	161 (71)*	208 (68)
Single	136 (60)	180 (59)
Employed	159 (71)	214 (71)
Currently smokes	75 (33)	94 (31)
Currently drinks alcohol	122 (54)	161 (53)
Mean age at sexual debut	18 (3)	17 (3)
Ever had sex with men	10 (4)	15 (5)
> 1 sexual partner in past 3 months	53 (23)	77 (25)
Consistent condom use with recent partner	143 (63)	190 (63)
On ART	144 (63)	197 (65)
Duration on ART, median months	31 (13–55)	33 (15–58)
CD4+ count, median cells/μL (*n* = 288)	443 (327–556)	445 (328–567)
Undetectable PVL (<40 copies/ml)	83 (39)	107 (54)
Circumcised	43 (19)	62 (20)
Anogenital warts present	36 (16)*	36 (12)
Accepted intra-anal swabbing	160 (70)	250 (82)
HPV detected on intra-anal swab (*n* = 227)	84 (37)	88 (39)
Anal cytology results (*n* = 242)^a^		
NILM	94 (41)	123 (51)
ASCUS	37 (16)	48 (20)
LSIL	50 (22)	70 (29)
HSIL	0 (0)	0 (0)

Of 304 genital swabs sent for analysis, 21 had invalid results. *: *p*-value < 0.05 for comparison of those with and without genital HPV infection. PVL, plasma viral load among those on antiretroviral therapy (ART). ^a^250 smears were sent for analysis, 8 had invalid results. *NILM* Negative for intraepithelial malignancy. *ASCUS* Atypical Squamous Cells of Undetermined Significance. *LSIL* Low-grade Squamous Intraepithelial Lesion. *HSIL* High-grade Squamous Intraepithelial Lesion

### Prevalence of HPV infection

**Fig. 1:**
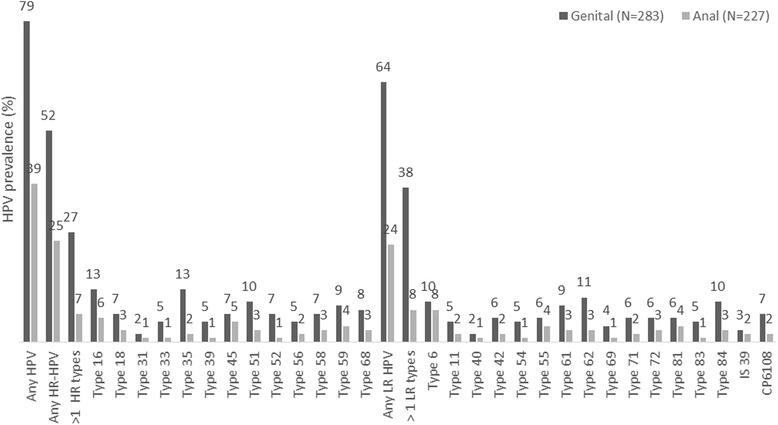
Prevalence of HPV infection by anatomical site

All 304 participants provided genital swabs. HPV DNA of any type was detected in 224 of the 283 valid genital swab samples (79%; Figure 1). Aside from being born in South Africa (71%), no other differences were detected in the characteristics of men with and without HPV DNA (Table 1). The median number of infections detected per genital swab was 2.0 (IQR, 1.0–4.0). HR-HPV was detected in 52% (*n* = 147) of samples. HPV 16 and 35 were the most frequently detected HR-HPV types (13% of men), and 18% had an HPV 16 and/or HPV 18 infection (51/283). Multiple HR-HPV infections (76/283) were observed in more than a quarter of men (27%), and 35% (51/147) of HR-HPV infections could be attributed to types 16 or 18. LR-HPV infections were more common than HR-HPV (64%, *n* = 182, *p* = 0.05) and 38% (108/283) of men had multiple LR-HPV infections. Among the men with LR-HPV, 16 (9%) also had at least one HR-HPV infection. The commonest LR-HPV types observed were types 62 (11%), 6 (10%) and 84 (10%).

The uptake of anal swabbing was high, with 82% (250/304) of men agreeing to the procedure. Of the 250 swabs taken, 227 had valid results (91%) and HPV DNA was detected in 39% (*n* = 88) of them (Figure 1)*.* In contrast to the genital swabs, the prevalence of HR-HPV infection in the anal canal (25%) was comparable to that of LR-HPV (24%) detected in these swabs. Fewer men had either multiple HR-HPV type (7%) or LR-HPV type (8%) infections in the anal canal compared to the genital swabs. Of the HR-HPV types, the most commonly detected types were 16 (6%), 45 (5%) and 59 (4%). Among all HR-HPV infections detected in the anal canal (*n* = 56), 43% were type 16 or 18. HPV types 6 (8%), 55 (4%) and 81 (4%) were the most common LR-HPV types detected. Together types 6 or 11 made up 46% (25/54) of all LR-HPV infections detected in the anal canal.

Results of both genital and anal swabs were available for 227 men, of these only 1% (*n* = 3) had no HPV infection, 58% (*n* = 132) had HPV infection of the genitalia only, 4% (*n* = 8) anal canal only and 37% (*n* = 84) had infection of both sites. A total of 168 men had an HR-HPV infection (either genital or anal canal), of these 33% had infection in both sites. Corresponding figures for LR-HPV were 177 and 29%. In terms of HPV genotypes among the 227 men with valid genital and anal swab results, the number who were infected with the same genotype in both sites (n) of all men with the genotype (N) were (i) HPV 16 (8/38) [21%] (ii) HPV 18 (2/21) [10%] (iii) HPV 6 (9/44) [21%] and (iv) HPV 11 (3/23) [13%].

### Prevalence and factors associated with abnormal anal cytology

**Fig. 2:**
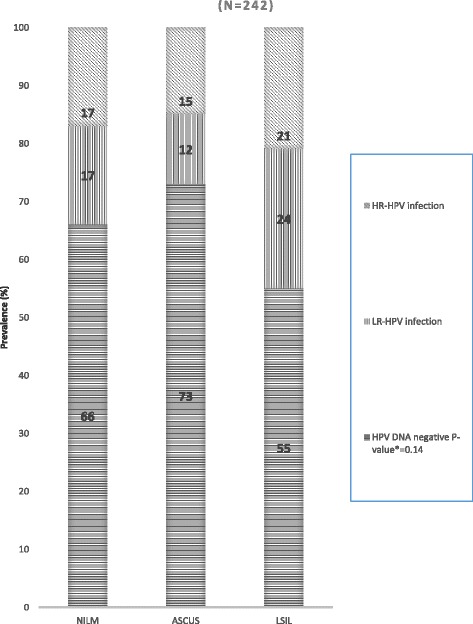
Prevalence of intra-anal HPV DNA infection by cytology grade. *P-value for the association between HR-HPV prevalence and cytology grade

Valid cytological results were available for 242 of the 250 anal smears (Table 1). Abnormalities were detected in half of the smears (49%). Among those with abnormal anal cytology results, 20% were graded as ASCUS and 29% as LSIL. No cases of ASC-H, HSIL or invasive cancer were detected. HR-HPV was detected more frequently in LSIL than in ASCUS (21% vs. 15%; Figure 2). In LSIL cases, type 16 was most prevalent 13% (*n* = 9), this was followed by, in order of decreasing frequency, HPV 45 (9%), HPV 59 (7%), HPV 18 (6%), HPV 68 (6%), HPV 31, 33, 35 & 58 all at 4%, HPV 51 & 56 all at 3%, and HPV 52 (1%). HPV 39 was not detected in this group (Additional file 1: Table S1).

We explored whether ART, participant’s health status and different HPV infections were associated with abnormal anal cytology (Table 2 and Additional file 2: Table S2). Current ART use was associated with an increased odds of abnormal anal cytology in models adjusting for age (adjusted OR = 2.26; 95%CI = 1.26–4.09; *p* = 0.007), while associations were not detected between abnormalities, and time since ART initiation, current CD4+ count and PVL. Intra-anal HPV infection was associated with abnormal anal cytology (Additional file 2: Table S2). Infection with more than one HR type (aOR = 2.39; 95%CI = 1.02–5.58; *p* = 0.04), and any alpha-9 types (aOR = 3.98; 95%CI = 1.42–11.16; *p* = 0.01) were strongly linked with an increased risk of abnormal cytology in age-adjusted models. Infection with type 16 was marginally associated with abnormal anal cytology (aOR = 3.30; 95%CI = 0.87–12.51; *p* = 0.08). No association was observed between presence of AGWs and abnormal anal cytology. After further adjusting for marital status, CD4+ count and duration on ART, no significant associations were observed between intra-anal HPV infection and abnormal cytology (Additional file 2: Table S2). Men with alpha-9 types had 3 times the odds of having abnormal cytology compared to those without the infection (OR = 3.08, 95%CI0.84–11.35; *p* = 0.09) and those with HPV type 16 had over 5.12 times the odds of abnormal cytology (OR = 5.12, 95%CI0.72–36.52; *p* = 0.10).

**Table 2:** The socio-behavioural, clinical and HIV-related factors associated with abnormal anal cytology

Characteristic	Abnormal anal cytology *N* = 118 n (%) or mean (SD)	M 1 (Crude) OR (95% CI)	*p*-value	M 2^a^ aOR (95%CI)	*p*-value
Born in SA	84 (72)	1.38 (0.80–2.37)	0.25	1.38 (0.79–2.38)	0.25
Single	85 (72)	1.76 (0.98–3.00)	0.06	1.76 (1.00–3.11)	0.05
Employed	83 (70)	0.86 (0.49–1.51)	0.60	0.86 (0.48–1.55)	0.62
Currently smokes	35 (30)	1.06 (0.61–1.85)	0.84	1.06 (0.61–1.85)	0.84
Currently drinks alcohol	63 (53)	1.23 (0.74–2.03)	0.43	1.23 (0.74–2.05)	0.42
Age at sexual debut	18 (3)	1.02 (0.94–1.10)	0.70	1.02 (0.94–1.10)	0.70
Ever had sex with men	8 (5)	1.60 (0.44–5.80)	0.48	1.60 (0.44–5.86)	0.47
> 1 sexual partner in past 3 months	25 (21)	0.90 (0.49–1.67)	0.75	0.90 (0.49–1.67)	0.74
Consistent condom use with recent partner	77 (65)	1.87 (0.86–4.08)	0.12	1.86 (0.86–4.12)	0.12
Anogenital warts present	10 (8)	0.67 (0.29–1.55)	0.34	0.67 (0.29–1.55)	0.35
On ART	88 (75)	2.08 (1.20–3.59)	0.009	2.26 (1.26–4.09)	0.007
Duration on ART (months)					
> 12	68 (58)	1		1	
6–12	16 (14)	1.13 (0.49–2.65)	0.77	1.01 (0.43–2.41)	0.97
< 6	3 (3)	0.47 (0.11–2.04)	0.31	0.41 (0.09–1.82)	0.24
CD4+ count (cells/μL)					
> 500	37 (31)	1		1	
351–500	41 (35)	1.65 (0.88–3.08)	0.12	1.65 (0.88–3.09)	0.12
200–350	21 (18)	1.03 (0.51–2.09)	0.94	1.03 (0.50–2.09)	0.94
< 200	12 (10)	2.27 (0.81–6.32)	0.12	2.26 (0.81–6.32)	0.12
Undetectable PVL (<40 copies/ml)	51 (43)	0.64 (0.38–1.10)	0.10	0.63 (0.36–1.09)	0.09
Controlled HIV disease^b^	33 (28)	1.38 (0.77–2.48)	0.28	1.35 (0.81–2.36)	0.30

Abnormal cytology included ASCUS and LSIL. M2^a^: Adjusted for age. ^b^: On ART for >6 months, CD4+ > 350 and undetectable PVL

### Association between LR HPV infections, ART and HIV disease, and AGWs

Twelve percent of all men had AGWs (*n* = 36; Table 1). Of these, 31% (*n* = 11) had intra-anal HPV infection whilst 28% (*n* = 10) had abnormal anal cytology (Table 3). Circumcision was associated with reduced odds of AGWs (aOR = 0.33; 95%CI = 0.09–1.13; *p* = 0.07). The presence of AGWs was influenced by several HIV-related factors. Shorter duration on ART was associated with increased odds of AGWs detection, and there was evidence of an inverse dose response relationship. Relative to being on ART for >12 months, being on ART for 6–12 months (aOR = 3.54; 95%CI = 1.29–9.72; *p* = 0.01) and <6 months (aOR = 6.90; 95%CI = 1.63–29.20; *p* = 0.009) increased the odds of AGWs. While HIV PVL was not associated with the detection of AGWs, having a lower CD4+ count was associated with a higher odds of having AGWs. Those with the lower CD4+ counts (i.e. <200 cells/μL) had the greatest risk for AGWs (aOR = 5.48; 95%CI = 1.60–18.78; *p* = 0.01, compared to those with CD4+ >500). Men who had evidence of HIV disease control (i.e. on ART for >6 months with undetectable PVL and CD4+ count >350 cells/μL) had a reduced likelihood for detection of AGWs (aOR = 0.22; 95%CI = 1.20–0.79; *p* = 0.02).

**Table 3:** The socio-behavioural, clinical and HIV-related factors associated with anogenital warts

Characteristic	AGW detected *N* = 36 n (%) or mean (SD)	M 1 (Crude) OR (95% CI)	*p*-value	M2 ^a^ OR (95%CI)	*p*-value
Born in SA	27 (75)	1.44 (0.65–3.20)	0.36	1.60 (0.71–3.58)	0.25
Marital status (Single)	29 (80)	2.00 (0.83–4.81)	0.12	1.71 (0.69–4.21)	0.24
Employed	26 (72)	1.20 (0.54–2.67)	0.66	1.02 (0.45–2.30)	0.97
Currently smokes	12 (33)	1.13 (0.54–2.38)	0.74	1.16 (0.55–2.44)	0.70
Currently drinks alcohol	20 (56)	1.18 (0.58–2.41)	0.65	1.09 (0.53–2.24)	0.82
Age at sexual debut	18 (3)	0.99 (0.89–1.11)	0.91	1.00 (0.89–1.13)	0.93
Ever had sex with men	1 (3)	0.52 (0.07–4.06)	0.53	0.48 (0.06–3.80)	0.49
> 1 sexual partners in past 3 months	8 (22)	0.89 (0.38–2.04)	0.78	0.82 (0.35–1.92)	0.65
Consistent condom use with recent partner	25 (69)	1.81 (0.52–6.31)	0.35	2.16 (0.61–7.70)	0.23
Circumcised	3 (8)	0.32 (0.10–1.09)	0.06	0.33 (0.09–1.13)	0.07
On ART	26 (72)	1.47 (0.68–3.19)	0.32	2.19 (0.94–5.07)	0.07
Duration on ART (months)					
> 12	14 (39)	1		1	
6–12	8 (22)	4.03 (1.50–10.81)	0.006	3.54 (1.29–9.72)	0.01
< 6	4 (11)	8.06 (1.94–33.50)	0.004	6.90 (1.63–29.20)	0.009
CD4+ count (cells/μL)					
> 500	7 (19)	1		1	
351–500	10 (28)	1.72 (0.63–4.71)	0.29	1.62 (0.58–4.49)	0.93
200–350	12 (33)	3.68 (1.36–9.95)	0.01	4.34 (1.57–12.07)	0.01
< 200	6 (17)	4.32 (1.32–14.25)	0.02	5.48 (1.60–18.78)	0.01
Undetectable PVL (<40 copies/ml)	11 (31)	0.65 (0.31–1.39)	0.26	0.77 (0.35–1.68)	0.51
Controlled HIV disease^b^	3 (8)	0.25 (0.08–0.85)	0.03	0.22 (0.12–0.79)	0.02
Cytological abnormalities	10 (28)	0.67 (0.29–1.55)	0.35	0.67 (0.29–1.59)	0.37

M2^a^: Adjusted for age. PVL plasma viral load. ^b^: On ART for >6 months, CD4+ > 350 and undetectable PVL

As shown in Additional file 3: Table S3, the detection of any LR-HPV infection was strongly associated with presence of AGWs in age-adjusted models (aOR = 41.28; 95%CI = 13.57–125.62; *p* < 0.001). MLWH with more than one LR-HPV type were also more likely to have AGWs (aOR = 4.14; 95%CI = 1.60–10.69; *p* = 0.003) than those with one LR-HPV type or less. Type-specific infections were also strongly associated with the presence of AGWs. Having type 6 (aOR = 3.97; 95%CI = 1.50–10.62; *p* = 0.006), type 55 (aOR = 3.10; 95%CI = 1.19–8.07; *p* = 0.02) or type 61 (aOR = 2.78; 95%CI = 1.28–6.32; *p* = 0.01) were all strongly associated with AGWs. After further adjusting for circumcision status, CD4+ count and duration on ART (Additional file 3: Table S3), similar associations with M2 were observed except that associations with types 55 and 61 became insignificant (effect sizes of associations were similar to M1 and M2). Type 11 was associated with AGWs in M3 only (aOR = 11.50, 95%CI = 1.10–119.18; *p* = 0.04).

## Discussion

In this study we estimated the prevalence of anogenital HPV infection and associated disease, their risk factors and uptake of anal swabbing using baseline data from a cohort of 304 MLWH. The prevalence of genital and anal HPV infection was relatively high at 79% and 39% respectively. This was matched by a 12% prevalence of AGW and almost half having anal cytological abnormalities.

The burden of genital HPV infection was consistent with findings from other studies among MLWH in South Africa, as well as in other settings [6, 42]. For example, the prevalence of genital HPV infection among 161 MLWH in Cape Town, South Africa was 77% [42]. Similarly, the levels of intra-anal HPV infection in this population of predominantly men who have sex with women mirrors the findings of other studies of anal infection (ranging between 36 and 59%) among MLWH without a history of receptive anal intercourse [43–45]. These infections could be due to non-sexual transfer of HPV between the male genitals and the anal canal which can occur via self-inoculation (i.e. direct transfer from genitals to the anus), contaminated fingers and other inanimate objects [46–49]. The fairly high concordance between genital and anal HPV infections observed in our study supports the concept of non-sexual transfer.

We showed that intra-anal swabs were acceptable to African MLWH, with the large majority agreeing to sampling. We did not observe any HSIL in this population of predominantly men who have sex with women who were in reasonably good health as evidenced by high a median CD4+ count, and the proportion of participants with controlled HIV disease. A considerable proportion of anal cytology specimens were, however, ASCUS or LSIL, which is reflective of prevalent HPV infection. In terms of the natural history of HPV infection, low-grade lesions (ASCUS and LSIL) represent the acute phase of infection with cellular proliferation [50], whilst HSIL is more representative of established disease (AIN). These findings are similar to other cohorts of MLWH which show that on average 50% of anal smears are NILM, 30–40% are LSIL and typically less than 5% are HSIL [51, 52].

It is worth noting that in the current study, being on ART was associated with cytological abnormalities. This could be because this study is cross sectional and we cannot comment on the temporal nature of events, but most likely patients with worse immune function are more likely to have anal disease and to have recently initiated onto therapy. However, the duration on ART, CD4+ count and virological control status were not linked with cytological abnormalities, and the association between ART and cytological abnormalities should be closely examined during longitudinal follow-up. Moreover, given that men infected with more than 1 HR type and at least one alpha-9 type (i.e. types 16, 31, 33, 35, 52 and 58) had a 2 to 4-fold-increased likelihood of having anal cytological abnormalities, it would be important to prospectively monitor men with these infections to better understand the factors that influence disease progression in this population and how ART interacts with these processes.

Levels of AGWs in our study were within the upper range of the AGW prevalence among men in Southern Africa, where studies have reported prevalence of between 2 and 14% [27, 53]. Men with genital HPV 6 infections were almost 4 times more likely to have prevalent AGWs in age-adjusted models, an almost identical odds ratio of this association that was noted in a study of 245 MLWH in Australia who have sex with men [54]. Overall, the study findings provide a compelling argument for the use of the HPV vaccination as primary prevention for AGWs in men, particularly those living with HIV. HPV vaccines are safe and immunogenic in HIV-positive individuals [55]. Despite the overall high HPV burden among HIV-infected individuals, the majority of them, both in this study and others, do not have infection with HPV types included in current HPV vaccines, and thus could benefit from vaccination. For example, the DNA prevalence of the vaccine preventable types 16 and 18 among HIV-positive women were 16% and 11% respectively in a study in Puerto Rico [56]. This means that more than 80% could potentially benefit from HPV vaccination as primary prevention. Moreover, rapidly accruing evidence also indicates that the vaccine is also beneficial as a secondary prevention strategy for the prevention of recurrent anal neoplasia and recurrent AGWs [57–59].

A lower CD4+ count was associated with an increased risk for AGWs, consistent with reports from other studies [26, 60]. This observation is primarily attributed to immune dysfunction, which impairs the clearance of HPV infections [24, 28]. In this study, a longer duration on ART (i.e. >12 months), and not the ART status per se, was associated with a reduced likelihood of prevalent AGWs. This is consistent with data showing that some time is required for immune reconstitution to occur before the clinical effects of ART are observed [61]. The multivariate analysis performed in the study (M3) illustrates the important role of age, marital status, duration on ART, CD4+ count with abnormal cytology as >1 HR type and alpha 9 types became insignificant in this model.

Limitations of the study include its cross-sectional design, which means that causality cannot be inferred about the associations observed. The absence of a sampling frame for the population made it hard to obtain a random, representative sample of the target population. We recruited from both community and healthcare settings in an attempt to increase the representativeness of the sample. Nevertheless, men in clinical settings and those in the community who accept to take part in a study may have different health seeking behaviours and medical conditions than other men. Plausibly, such differences could both under- or overestimate HPV prevalence and disease, and thus caution needs to be applied when interpreting and generalising the prevalence estimates. Also, the low numbers of MSM in the study diminished the ability to detect associations between this exposure and the study outcomes. Having had sex with men may have been under-reported, given that the behaviour remains stigmatised. It is, however, also possible that under-reporting did not occur as other evidence also indicates that HPV infection is common in men who have sex with women and who do not report receptive anal intercourse [2, 62].

In terms of methods, visual inspection for AGWs detection was done without histological confirmation, making it possible that other conditions (e.g. penile intraepithelial neoplasia) could have been misclassified as warts. Similarly, we used conventional anal smears which could have misclassified cytological outcomes. We did not have access to high resolution anoscopy (HRA) and guided biopsy, the gold standard for pathological diagnosis. The study, however, used the Roche Linear Array for HPV DNA testing and genotyping, which has greater accuracy and specificity than other commonly used assays, such as INNO-LiPA and reverse hybridization [63]. However, with use of Roche Linear Array, the potential HR types: 26, 53, 66 and HR types 73, 82 may be missed as these are classified as LR in that test, while INNO-LiPA classifies them as potential HR and HR types respectively. Nevertheless, despite these limitations, the study does contribute important data on the prevalence of anogenital HPV infection and associated disease, including the impact of ART among MLWH. Longitudinal data will more fully explore the incidence, persistence, clearance or regression of anogenital HPV infection and associated disease, and the impact of ART so as to inform guidelines on preventing and treating HPV associated disease in this key population.

## Conclusions

In this African inner-city setting among MLWH who are predominantly men who have sex with women, anogenital HPV infection is very common, associated disease is also relatively frequent, but no cases of severe anal dysplasia were noted. The study does not support the use of screening for anal dysplasia in this population since we did not detect any high-grade anal lesions, and as there would be substantial laboratory and programmatic challenges involved in such a programme. HPV vaccination would be of benefit for the primary and secondary prevention of both AGWs and HR-HPV infections, even though it is still not clear whether this translates into a reduction of anal disease.

## Additional files

Additional file 1: Table S1. Distribution of the HPV genotypes according to the cytological diagnosis. (DOC 66 kb)

Additional file 2: Table S2. Associations between intra anal HPV infection and abnormal cytology. (DOC 57 kb)

Additional file 3: Table S3. Associations between genital HPV infection and anogenital warts. (DOC 57 kb)

## Abbreviations

AGWs: anogenital warts
AIN: anal intraepithelial neoplasia
ART: antiretroviral therapy
ASC-H: atypical squamous cells-high grade lesions cannot be ruled out
ASCUS: atypical squamous cells of undetermined significance
HPV: human papillomavirus
HR: high-risk
HRA: High Resolution Anoscopy
HSIL: high-grade squamous intraepithelial lesion
IQR: interquartile range
LMICs: low- and middle-income countries
LR: low-risk
LSIL: low-grade squamous intraepithelial lesion
MLWH: men living with HIV
MSM: men who have sex with men
NHLS: National Health Laboratory Service
NILM: negative for intraepithelial malignancy
pHR: possible High Risk
PVL: plasma viral load
SD: standard deviation
SSA: sub-Saharan Africa
STI: sexually transmitted infection
