# Supplementary material for: Prevalence of anogenital HPV infection, related disease and risk factors among HIV-infected men in inner-city Johannesburg, South Africa: baseline findings from a cohort study
Source: BMC Public Health. 2017 Jul 4;17(Suppl 3):425. doi: 10.1186/s12889-017-4354-0 (PMC5498864; doi:10.1186/s12889-017-4354-0)
Supplement: Supplementary file 1 — Distribution of the HPV genotypes according to the cytological diagnosis. (DOC 66 kb) [file 12889_2017_4354_MOESM1_ESM.doc]

**Additional file 1 Table S1: Distribution of the HPV genotypes according to the cytological diagnosis**

| **GENOTYPE** | **NILM (N=123)** | | **ASCUS (N=48)** | | **LSIL (N=70)** | |
| --- | --- | --- | --- | --- | --- | --- |
| **HR Types** | **n** | **%** | **n** | **%** | **n** | **%** |
| HPV 16 | 3 | 2 | 0 | 0 | 9 | 13 |
| HPV 18 | 3 | 2 | 1 | 2 | 4 | 6 |
| HPV 31 | 0 | 0 | 0 | 0 | 3 | 4 |
| HPV 33 | 0 | 0 | 0 | 0 | 3 | 4 |
| HPV 35 | 0 | 0 | 1 | 2 | 3 | 4 |
| HPV 39 | 1 | 1 | 0 | 0 | 0 | 0 |
| HPV 45 | 6 | 5 | 0 | 0 | 6 | 9 |
| HPV 51 | 3 | 2 | 1 | 2 | 2 | 3 |
| HPV 52 | 0 | 0 | 0 | 0 | 1 | 1 |
| HPV 56 | 1 | 1 | 1 | 2 | 2 | 3 |
| HPV 58 | 2 | 2 | 1 | 2 | 3 | 4 |
| HPV 59 | 4 | 3 | 0 | 0 | 5 | 7 |
| HPV 68 | 0 | 0 | 0 | 0 | 4 | 6 |
| **LR types** | **n** | **%** | **n** | **%** | **n** | **%** |
| HPV 6 | 11 | 9 | 2 | 4 | 4 | 6 |
| HPV 11 | 1 | 1 | 0 | 0 | 2 | 3 |
| HPV 42 | 3 | 2 | 0 | 0 | 1 | 1 |
| HPV 55 | 5 | 4 | 0 | 0 | 2 | 3 |
| HPV 61 | 5 | 4 | 0 | 0 | 3 | 4 |
| HPV 72 | 3 | 2 | 0 | 0 | 2 | 3 |
| HPV 81 | 4 | 3 | 1 | 2 | 5 | 7 |
| HPV 84 | 2 | 2 | 2 | 4 | 3 | 4 |

NILM, Negative for intraepithelial malignancy. ASCUS, Atypical Squamous Cells of Undetermined Significance. LSIL, Low-grade Squamous Intraepithelial Lesion. HR high risk. LR low risk.
