# Supplementary material for: Prevalence of anogenital HPV infection, related disease and risk factors among HIV-infected men in inner-city Johannesburg, South Africa: baseline findings from a cohort study
Source: BMC Public Health. 2017 Jul 4;17(Suppl 3):425. doi: 10.1186/s12889-017-4354-0 (PMC5498864; doi:10.1186/s12889-017-4354-0)
Supplement: Supplementary file 2 — Associations between intra anal HPV infection and abnormal cytology. (DOC 57 kb) [file 12889_2017_4354_MOESM2_ESM.doc]

**Additional file 2: Table S2: Associations between intra anal HPV infection and abnormal cytology**

| **Characteristic** | **Abnormal anal cytology**  **N=118**  **n (%) or mean (SD)** | **M 1 (Crude)**  **OR (95% CI)** | **p-value** | **M 2***  **aOR (95%CI)** | **p-value** | **M3****  **aOR (95%CI)** | **p-value** |
| --- | --- | --- | --- | --- | --- | --- | --- |
| Intra-anal HPV |  |  |  |  |  |  |  |
| Any HR Type | 27 (26) | 1.23 (0.62-2.43) | **0.55** | 1.23 (0.62-2.43) | 0.55 | 0.87 (0.32-2.40) | 0.79 |
| > 1 HR type | 18 (15) | 2.40 (1.03-5.59) | **0.04** | 2.39 (1.02-5.58) | **0.04** | 0.87 (0.33-2.33) | 0.79 |
| 16 and 18 | 13 (11) | 2.41 (0.89-6.58) | 0.08 | 2.42 (0.89-6.58) | 0.08 | 2.44 (0.63-9.46) | 0.19 |
| Alpha-7 types | 10 (8) | 0.94 (0.38-2.31) | 0.90 | 0.94 (0.38-2.30) | 0.98 | 0.73 (0.25-2.16) | 0.57 |
| Alpha-9 types | 17 (14) | 3.97 (1.42-11.15) | **0.009** | 3.98 (1.42-11.16) | **0.01** | 3.08 (0.84-11.35) | 0.09 |
| Type 16 | 9 (8) | 3.30 (0.87-12.51) | 0.08 | 3.30 (0.87-12.51) | 0.08 | 5.12 (0.72-36.52) | 0.10 |
| Type 18 | 5 (4) | 1.78 (0.41-7.58) | 0.44 | 1.78 (0.42-7.61) | 0.44 | 0.94 (0.14-6.12) | 0.95 |
| Type 45 | 6 (5) | 1.04 (0.33-3.34) | 0.94 | 1.03 (0.32-3.32) | 0.95 | 1.05 (0.25-4.40) | 0.94 |
| Type 51 | 3 (3) | 1.04 (0.21-5.28) | 0.95 | 1.05 (0.20-5.38) | 0.95 | 0.96 (0.14-6.55) | 0.97 |
| Type 56 | 3 (3) | 3.18 (0.33-31.04) | 0.32 | 3.27 (0.33-32.13) | 0.31 | 0.79 (0.07-9.51) | 0.85 |
| Type 58 | 4 (4) | 2.12 (0.38-11.81) | 0.39 | 2.12 (0.38-11.81) | 0.39 | 1.50 (0.24-9.50) | 0.66 |
| Type 59 | 5 (4) | 1.32 (0.34-5.03) | 0.68 | 1.32 (0.35-5.03) | 0.68 | 0.94 (0.19-4.58) | 0.94 |

Abnormal cytology included ASCUS and LSIL. M2*: Adjusted for age. M3**: Adjusted for age, marital status, duration on ART and CD4+ count. Alpha-7 includes: HPV 18, 39, 45 and 59. Alpha-9 includes: HPV 16, 31, 33, 35, 52 and 58.
