# Supplementary material for: Prevalence of anogenital HPV infection, related disease and risk factors among HIV-infected men in inner-city Johannesburg, South Africa: baseline findings from a cohort study
Source: BMC Public Health. 2017 Jul 4;17(Suppl 3):425. doi: 10.1186/s12889-017-4354-0 (PMC5498864; doi:10.1186/s12889-017-4354-0)
Supplement: Supplementary file 3 — Associations between genital HPV infection and anogenital warts. (DOC 57 kb) [file 12889_2017_4354_MOESM3_ESM.doc]

**Additional file 3: Table S3: Associations between genital HPV infection and anogenital warts**

| **Characteristic** | **AGW detected N=36**  **n (%) or mean (SD)** | **M 1 (Crude)**  **OR (95% CI)** | **p-value** | **M2 ***  **OR (95%CI)** | **p-value** | **M3 ****  **OR (95%CI)** | **p-value** |
| --- | --- | --- | --- | --- | --- | --- | --- |
| Genital HPV |  |  |  |  |  |  |  |
| Any LR type | 32 (89) | 42.67 (14.06-129.49) | **<0.001** | 41.28 (13.57-125.62) | **<0.001** | 25.33 (6.54-98.19) | **<0.001** |
| > 1 LR type | 5 (14) | 3.39 (1.36-8.43) | **0.009** | 4.14 (1.60-10.69) | **0.003** | 3.85 (1.21-12.21) | **0.02** |
| 6 and 11 | 4 (11) | 4.20 (1.66-10.61) | **0.02** | 4.59 (1.78-11.80) | **0.02** | 8.64 (2.45-30.50) | **0.002** |
| 6 | 23 (64) | 3.79 (1.44-9.97) | **0.007** | 3.97 (1.50-10.62) | **0.006** | 7.36 (2.02-26.80) | **0.002** |
| 11 | 14 (39) | 5.18 (0.83-32.09) | 0.08 | 5.09 (0.80-32.30) | 0.08 | 11.50 (1.10-119.18) | **0.04** |
| 42 | 6 (17) | 2.62 (0.97-7.07) | **0.06** | 2.36 (0.86-6.45) | 0.09 | 2.59 (0.17-39.67) | 0.50 |
| 55 | 7 (19) | 2.99 (1.17-7.68) | **0.02** | 3.10 (1.19-8.07) | **0.02** | 2.09 (0.33-13.01) | 0.43 |
| 61 | 10 (28) | 2.84 (1.25-6.42) | **0.01** | 2.78 (1.28-6.32) | **0.01** | 3.22 (0.60-17.30) | 0.17 |
| 72 | 6 (17) | 2.21 (0.82-5.94) | 0.12 | 1.93 (0.13-5.26) | 0.93 | 0.78 (0.07-8.55) | 0.84 |
| 81 | 4 (11) | 1.39 (0.45-4.31) | 0.56 | 1.39 (0.45-4.34) | 0.56 | 0.83 (0.140-4.88) | 0.84 |
| 84 | 8 (22) | 1.68 (0.71-3.95) | 0.24 | 1.67 (0.70-3.92) | 0.25 | 1.88 (0.38-9.25) | 0.44 |

Abnormal cytology included ASCUS and LSIL. M2*: Adjusted for age. M3**: Adjusted age, circumcision status, duration on ART and CD4+ count.
